# Comments on “Diet quality in relation to the risk of hypertension among Iranian adults: cross-sectional analysis of Fasa PERSIAN cohort study”

**DOI:** 10.1186/s12937-022-00797-7

**Published:** 2022-06-21

**Authors:** Saeed Ghobadi, Alireza Jafari

**Affiliations:** 1 Non-Communicable Diseases Research Center, Alborz University of Medical Sciences, Karaj, Iran; 2 Physiology Research Center, Kerman University of Medical Sciences, Kerman, Iran

Dear editors,

We enthusiastically read the research of Motamedi et al. [1] that was published in the valuable Nutrition Journal. This cross-sectional study used the data of the Fasa Cohort Study as a branch of the Prospective Epidemiological Research Study in Iran (PERSIAN) cohort. The authors presented associations between the diet qualities, assessed by Mediterranean dietary score, dietary diversity score, healthy eating index-2015, and diet quality index-international with the risk of hypertension in an Iranian population. However, there are some concerns regarding the methodology and interpretation of the present study.

Firstly, in the dietary intake assessment part of the methods, the authors used a block food-frequency questionnaire (FFQ) to assess the diet qualities of their study population, but this FFQ was not validated for use in the Iranian population. The authors cited reference [25] to support the validity of **125**-item food frequency questionnaires (FFQ); however, the study conducted by Willett et al. [2] was **61**-item FFQ, and their findings should be interpreted with caution. Therefore, this study could have been strengthened by providing further information on the validity of the FFQ to use in this sample of the Iranian population.

Secondly, binary logistic regression analysis was used to identify the relationship between dietary quality indices and the risk of hypertension, but it has not been clarified how the confounders were selected for building the models. It is expected that crude effect size in multivariable models changes by at least 10% after adjusting covariates [3]. In addition, it is mentioned that independent variables with a P-value less than 0.2 should be considered for the multivariable analysis [4]. Also, we wonder why some confounders such as drugs and other agents affecting blood pressure were not controlled.

Finally, although daily energy intake has been considered the main confounders in all adjusted models, the authors have not presented the distribution of energy intake in the article. We found that in another article [5] has been published on the same population, the mean of daily energy intake was higher than 3500 and 2800 (kcal/day) in men and women, respectively. Therefore, when comparing the calculated energy intakes with other articles on the Iranian population [6, 7], energy intakes are far too high in respondents. We believe that the average energy intake in this study may be over-reported.

## Data Availability

Not applicable.
